# Transcriptional and Post-Translational Regulation of Plant bHLH Transcription Factors during the Response to Environmental Stresses

**DOI:** 10.3390/plants12112113

**Published:** 2023-05-26

**Authors:** Yasmina Radani, Rongxue Li, Harriet Mateko Korboe, Hongyu Ma, Liming Yang

**Affiliations:** 1 State Key Laboratory of Tree Genetics and Breeding, Co-Innovation Center for Sustainable Forestry in Southern China, College of Biology and the Environment, Nanjing Forestry University, Nanjing 210037, China; radani.yasmina@gmail.com (Y.R.); lirongxue@njfu.edu.cn (R.L.); harrietmatekokorboe27@gmail.com (H.M.K.); 2 College of Plant Protection, Nanjing Agricultural University, Nanjing 210095, China

**Keywords:** bHLH transcription factors, abiotic stress, transcriptional regulation, post-translational regulation

## Abstract

Over the past decades, extensive research has been conducted to identify and characterize various plant transcription factors involved in abiotic stress responses. Therefore, numerous efforts have been made to improve plant stress tolerance by engineering these transcription factor genes. The plant basic Helix–Loop–Helix (bHLH) transcription factor family represents one of the most prominent gene families and contains a bHLH motif that is highly conserved in eukaryotic organisms. By binding to specific positions in promoters, they activate or repress the transcription of specific response genes and thus affect multiple variables in plant physiology such as the response to abiotic stresses, which include drought, climatic variations, mineral deficiencies, excessive salinity, and water stress. The regulation of bHLH transcription factors is crucial to better control their activity. On the one hand, they are regulated at the transcriptional level by other upstream components; on the other hand, they undergo various modifications such as ubiquitination, phosphorylation, and glycosylation at the post-translational level. Modified bHLH transcription factors can form a complex regulatory network to regulate the expression of stress response genes and thus determine the activation of physiological and metabolic reactions. This review article focuses on the structural characteristics, classification, function, and regulatory mechanism of bHLH transcription factor expression at the transcriptional and post-translational levels during their responses to various abiotic stress conditions.

## 1. Introduction

**Figure 1:**
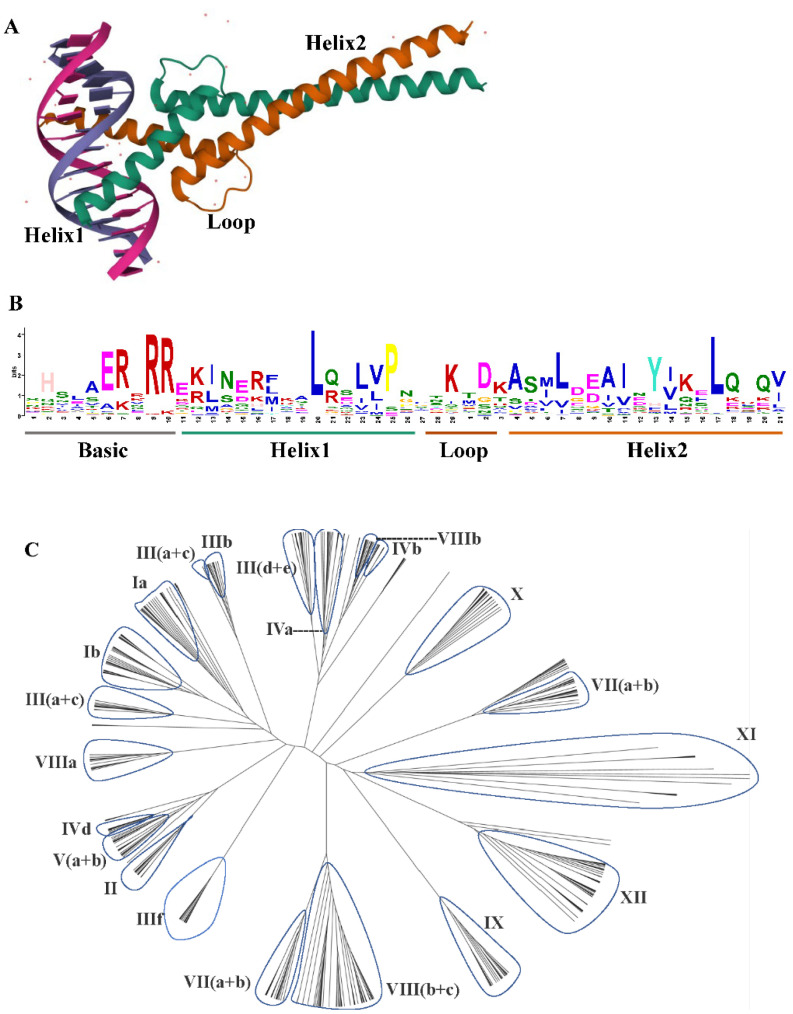
(**A**) The crystal structure of bHLH domain heterodimer bound to the DNA. (**B**) The sequence logo of the bHLH domain. The overall height of each stack represents the conservation of the sequence at that position. Each color of the letters represents a type of amino acid residue. (**C**) The phylogenetic tree analysis of *bHLH* gene family in *Oryza sativa* [monocot]; *Arabidopsis thaliana* [eudicot]; *Selaginella moellendorffii* [lycophyte]; *Physcomitrella patens* [moss]; *Volvox carteri*; *Chlamydomonas reinhardtii*; *Chlorella vulgaris*; *Ostreococcus tauri*; and *Cyanidioschyzon merolae* was constructed using MEGA7.0 with the JTT method and 1000 replicates. Then, the trees were visualized using Figtree. Group names were marked outside the circle. The protein sequences were downloaded from the report in 2010 [1].

The basic Helix–Loop–Helix (bHLH) Transcription Factors (TF) are a large and diverse family of transcription factors characterized by a highly conserved bHLH domain [1]. The bHLH transcription factor conservatively contains two connected subregions, namely the basic region (b), which is an essential DNA-binding region, and the HLH region, which consists of 40–50 amino acid residues and participates in homodimerization or heterodimerization [2] (Figure 1A,B). The amino acid sequences outside the bHLH region are divergent, even in closely related proteins from the same species. These short conserved amino acid motifs are commonly present in related plant bHLH proteins and are generally conserved within each subfamily [3,4].

The bHLH motif was first discovered in the murine transcription factors E12 and E4 [5]. Since then, several *bHLH* genes have been discovered, providing an initial classification of animals that divided bHLH transcription factors into six subgroups (A to F) based on their protein sequences, differences in bHLH domains, and comparisons of the functions of different family members [6,7]. Furthermore, outside of mammals, multiple classifications of 15 to 25 subfamilies based on bHLH and non-bHLH motifs have been obtained [3,4,8]. As more species were included, the most recent classification allows them to be divided into 26 subgroups [1], reflecting a deep evolutionary relationship in plants. In addition, phylogenetic analysis of several atypical bHLH proteins extended the number of subfamilies to 32 [8].

Maize (*Zea mays* L.) was the first plant species in which the bHLH superfamily was first identified. Subsequently, 162 *bHLH* genes were identified in the model plant *Arabidopsis thaliana* [9], 167 in rice (*Oryza sativa* L.) [4] and 202 *bHLH* genes in *Poplar* [10]. The number of characterized and identified *bHLH* genes in plants has increased, showing their extensive and diverse functional involvement; 100 *PmbHLH* genes were identified in *Prunus mume* [11], 102 *bHLH* genes in walnut (*Juglans regia* L.) [12], 212 *MibHLH* genes in mango (*Mangifera indica* L.) [13], 37 *SsbHLH* genes in sugarcane (*Saccharum spontaneum*) [14], 110 *IbbHLHs* in sweetpotato (*Ipomoea batatas* (L.) Lam.) [15], 118 *bHLH* genes were identified in melon [16], 107 *CabHLHs* were identified in *Capsicum annuum* [17], and 85 bHLH proteins (GbbHLH) were obtained from *Ginkgo biloba* [18]. *bHLH* gene families are widespread in plants and have demonstrated their essential roles in various biological processes involved in normal plant growth and development [1], flowering [19], and metabolic biosynthesis, including anthocyanin [20]. Therefore, many of them have a role and regulatory function in signal transduction [21,22] and gene expression in response to abiotic stresses such as salinity, drought, low temperature, and nutrient deficiency [23,24].

Many research studies have paid close attention to regulatory genes, including bHLH TFs, which play essential roles in multiple abiotic stress responses by regulating the expression of a wide range of downstream stress-responsive genes by interacting with the specific cis-elements in their promoter region [25]. Therefore, genetically modifying the expression of TFs can strongly affect plant stress tolerance as it mimics or enhances stress signals to simultaneously regulate many downstream stress-responsive genes. In recent years, several mechanisms of regulation and tolerance of bHLH TF to abiotic stress in model and non-model plants have been revealed, providing a better and more detailed explanation of their intervention under specific stress conditions. *bHLH*s are involved in various functional gene approaches to significantly enhance stress tolerance in plants. They can be activated under multiple stresses and play an essential role in abiotic stress responses by regulating a wide range of downstream stress-responsive genes [26]. Meanwhile, bHLH TFs themselves undergo various modifications at the post-translational level, such as ubiquitination [27], phosphorylation [28], and sumoylation [29], thereby forming a regulatory complex network to modulate the expression of *bHLH* stress-responsive genes.

Both transcriptional and post-translational modifications contribute to this plant stress response regulatory function. In this review, we focused on the structure and gene classification of bHLH TFs, as well as recent studies on their expression and the different mechanisms of transcriptional and post-translational regulation under different abiotic stress conditions.

## 2. Structure and Gene Family of bHLH TFs

The bHLH TF family, known for its conserved bHLH domain [30], is considered the second largest gene family after the MYB family gene and is found in the majority of eukaryotes. With a total of ~60 amino acids, the bHLH transcription factor is divided into two functionally distinct regions. On the one hand, there is the primary region, which is located in the N-terminal domain and contains a total of 15 to 20 amino acids, including basic amino acid residues, and is responsible for DNA binding, with certain conserved amino acids responsible for recognition of the E-box, a hexanucleotide consensus sequence in DNA (5-CANNTG-3). Other residues, on the contrary, are specific for a different E-box region in DNA (e.g., the G-box [5-CACGTG-3]) in target genes [25,31]. According to Toledo-Ortiz et al., *bHLH* genes can be divided into E-box or non-E-box binders and DNA-binding or non-DNA-binding proteins. The Helix–Loop–Helix (HLH) region, on the other hand, is found in the C-terminal domain and is composed of hydrophobic residues rearranged into two amphipathic regions connected to a loop region forming a hydrophobic ring (Figure 1B). They are involved in homo- or heterodimerization, as shown in Figure 1A, and thus participate in protein–protein interaction and gene expression control [2]. The dimer structure is stabilized by the hydrophobic amino acids Isoleucine (I), Leucine (L), and Valine (V) in conserved positions in the bHLH domain [30].

Outside of the bHLH domain, bHLH proteins show little to nonexistent conservation [3]. However, some groups of bHLH show some conserved domains outside of bHLH, most of which have been previously characterized in animals. For example, there is the highly conserved Leucine Zipper (LZ) motif adjacent to the second helix of the bHLH domain, which is predicted to adopt a coiled-coil structure, allowing protein dimerization. The PAS domain, the orange domain, the WRPW motif, and the COE domain are other domains also found in bHLH proteins [6,32]. The bHLH motif was first identified by Massari and Murre in 1989. Then, with more identified bHLH proteins, the first classification of different family members of animal bHLH TFs was performed using only the bHLH motif. This classification led to the selection of four distinct groups based on amino-acid patterns and E-box-binding specificity [7]. This classification separated bHLH proteins into classes A, B, C, and D [7].

Using the entire protein sequence and not just the bHLH domain allowed the inclusion of additional domains associated with many other proteins (e.g., Zip, Orange, and PAS) that may play important roles in protein dimerization and DNA binding. Therefore, the classification was extended to include two additional groups (E and F) [6,33]. The characterized bHLH proteins have been restricted to animals. It has been suggested that the ancestral plant bHLH sequence was a group B protein present in early eukaryote evolution, from which bHLHs of different lineages independently evolved [6,7] and function in transcriptional regulation associated with various diverse functions in plants including anthocyanin biosynthesis, phytochrome signaling, globulin expression, fruit dehiscence, and carpel and epidermal development [3]. Furthermore, some studies have aimed to divide the large bHLH family into smaller subgroups based on their sequence homology [1,3,8]. In 2003, Heim et al. compared the genetic structure, number of introns, and conservative motifs of 133 *bHLH* isolates from *Arabidopsis* and rearranged them into 12 subgroups. Since then, Pires and Dolan have divided bHLH into 26 subgroups by phylogenetic analysis, using a total of 544 *bHLH* genes from 9 land plants and algae [1] (Figure 1C). Meanwhile, Carretero-Paulet et al. added 29 to the original 133 *Arabidopsis bHLH* and classified them into 32 subfamilies by phylogenetic analysis, intron pattern, and conservative protein motif structure using 9 plant species [8].

Mainly, members of the same bHLH subfamily generally share similar gene structure and the same non-bHLH conserved motifs, such as a Leucine Zipper (LZ) domain (shared between IVb subfamilies and between IVc subfamilies) [7]. There is also an ACT domain, which is a ligand-binding regulatory domain found in a diverse group of proteins, mainly metabolic enzymes. The occurrence of the ACT domain in some proteins from different bHLH subfamilies suggests that the ACT bHLH association occurred multiple times, possibly through domain shuffling processes, suggesting that domain shuffling processes may play a small role in the evolution of *bHLH* genes in plants [1]. Members of the same plant bHLH subfamily are also often involved in the same biological processes, such as subgroup Va, which is involved in brassinosteroid signaling [34], and the Active Phytochrome Binding (APB) motif, encoded in several proteins of subfamily VII (a + b), which mediate the binding of multiple *A. thaliana* bHLH proteins to phytochrome B [1,35].

**Figure 2:**
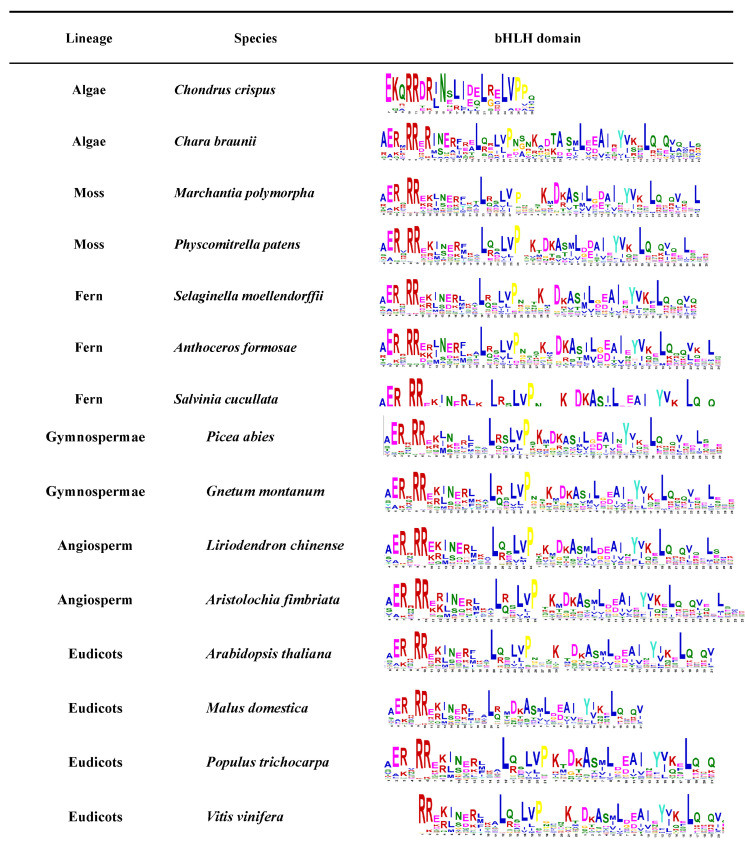

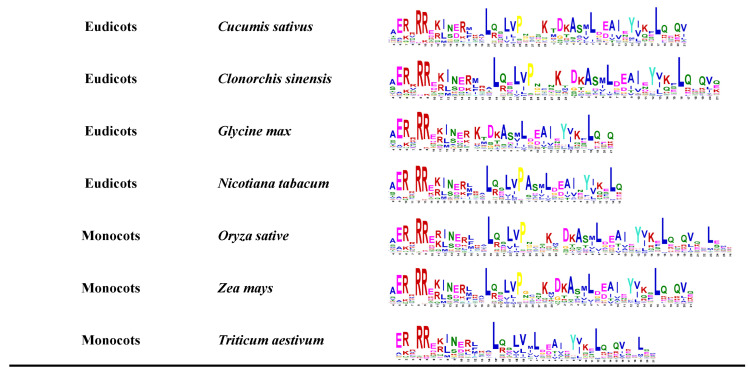
The bHLH domain conservative motif analysis expressed in different plants. Conservative domain motifs are used in MEME online web analytics. The bHLH protein sequences of these species were downloaded from the JGI and NCBI databases.

Plant bHLH diversity was already present in the early land plants before the moss and vascular plant lineages separated over 440 million years ago. Many current bHLH interactions also occurred in early land plants and were essentially conserved in the major plant groups [1]. The expansion of this family is closely related to plant evolution and diversity, not only in higher plants but also in lower plants such as algae, lichens, and mosses or non-plants such as mycobacteria [1]. The availability of a large number of land plants and algal genomes allowed further analysis of the conserved motifs of the *bHLH* gene family throughout the photosynthetic plant spectrum (Figure 2). Different numbers of members of the *bHLH* gene family have been identified in different species by searching for proteins containing the bHLH domain. The *bHLH* gene family was found to originate from Rhodophyta. The conservation of the bHLH conservative domain in different species was further predicted by MEME (Figure 2). According to the conservative motif, the bHLH domain will evolve to be more complete as species evolve. The evolution of the *bHLH* gene family also provides insights regarding the evolution of green algae into flowering plants through their adaptation to environmental changes [8]. Specifically, the genome-wide analysis of *bHLH* gene families from different species will help us to better understand the biological function, evolutionary origin, and expansion outcome of *bHLH* genes.

## 3. Regulation of bHLH TFs in Plant Stress Response

**Figure 3:**
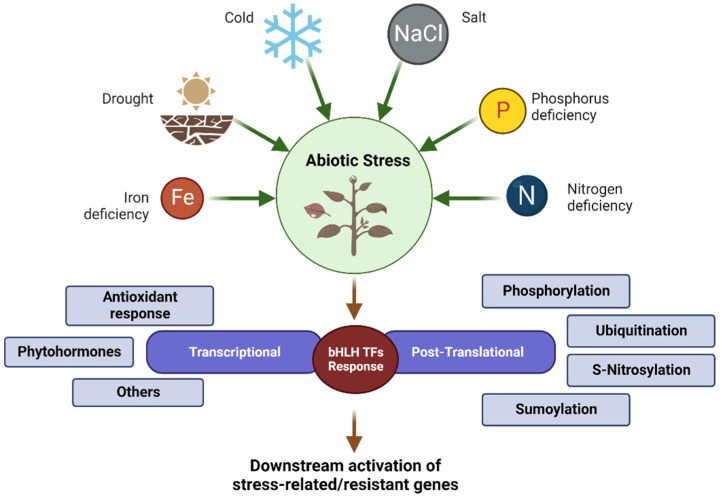
Summary of the different types of bHLH TFs responses to abiotic stresses in plants.

Previous research has revealed that the bHLH TF family can participate in the regulation of abiotic stresses such as drought, high salinity, low temperature, and nutrient deficiencies through transcriptional and post-translational modifications [36,37,38,39,40,41]. Transcriptome is commonly used in abiotic stress studies. Transcriptome technology can be used to identify key nodes or genes for stress resistance at the transcriptional regulatory level for subsequent research. In order to reveal the potential roles of bHLH members in various stress responses in different species, most functional signaling pathways of bHLH TF regulations were identified (Table 1 and Figure 3).

## 4. Drought Stress Response

One of the major environmental issues that reduces plant growth and development is drought. Drought-induced yield declines have been reported for many crop species, which depend on the severity and duration of the stress period and are associated with a decline in crop yield productivity [118]. Abscisic acid (ABA) is an important phytohormone that plays an important role in abiotic stress tolerance, whose main function appears to be the regulation of plant water balance and osmotic stress tolerance. Plant bHLH transcription factors generally respond to drought stress through both ABA-dependent and ABA-independent regulatory systems [119,120,121].

**Figure 4:**
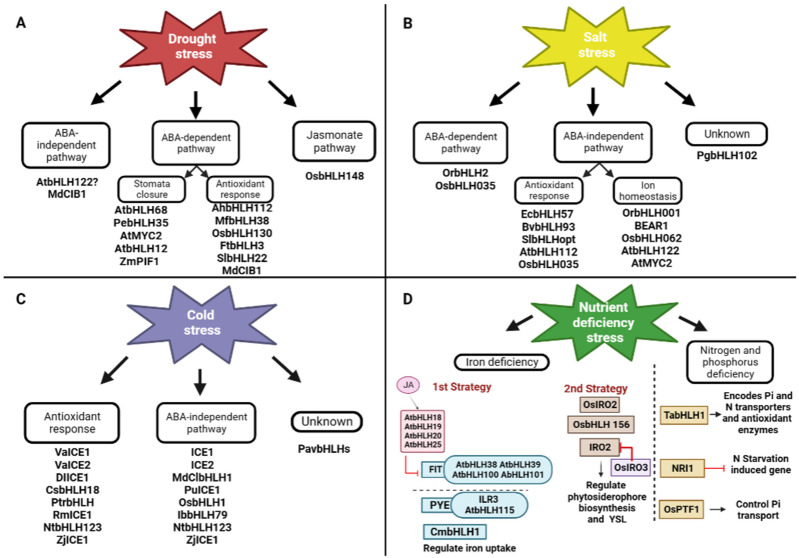
Transcriptional regulations of bHLH TF under abiotic stresses. (**A**) For drought stress; (**B**) for salt stress; (**C**) for cold stress; and (**D**) for nutrient deficiency stress.

In peanuts (*Arachis hypogaea*), AhbHLH112 responds to drought stress by regulating antioxidant gene-mediated reactive oxidative species (ROS) scavenging or ABA-dependent pathways as, under drought stress, ABA accumulates, and blocks Ca2+ influx and membrane proton pumps, resulting in plasma membrane depolarization and stomatal closure, which is required to reduce water loss and maintain high water potential. Under drought stress, AhbHLH112 is induced and could activate antioxidant genes and promote ROS scavenging (POD) [42]. *MfbHLH38* also acts positively on plant defenses via the ABA-dependent signaling pathway and enhances the ROS scavenging system, thereby reducing oxidative damage under stress [40]. Drought stress can also induce the accumulation of OsbHLH130, which activates *OsWIH2* expression, and the latter improves rice drought tolerance by reducing the water loss rate and ROS accumulation [43]. *FtbHLH3* responds to drought stress by increasing photosynthetic efficiency and upregulating the expression of critical genes in the ABA signaling pathway, the proline biosynthetic pathway, the ROS scavenging system, and the drought-responsive pathway [44]. *SlbHLH22* also improved tomato plant stress resistance by inducing the expression of genes involved in flavonoid biosynthesis. Flavonoids can enhance plant tolerance to drought and salt stress due to their ability to scavenge superoxides, peroxides, and free radicals generated during stress through ABA biosynthesis and the ROS scavenging pathway, leading to stomatal closure, increased proline content, and enhanced CAT, POD, and SOD activities with reduced ROS accumulation, resulting in an improved tolerance under abiotic stress [45]. ZmPIF1 is a positive regulator of root development, ABA synthesis, signaling pathways, and drought tolerance. It was found that ZmPIF1 binds to the G-box element in the promoters of NCED, CBF4, ATAF2/NAC081, NAC30, and other transcription factors and positively regulates their expression [46]. Cryptochrome-interacting bHLH1 (MdCIB1), through ABA-dependent and ABA-independent signaling pathways, regulates ABA signal transduction, antioxidant system, osmotic balance, and expression levels of stress-related genes [47]. AtbHLH68 may positively respond to drought stress through ABA signaling and by regulating ABA homeostasis in *Arabidopsis* [48]. *PebHLH35* in transgenic *Arabidopsis* confers drought tolerance by reducing stomatal density, opening, transpiration rate, and water loss, and increasing the chlorophyll content and photosynthesis rate [49]. AtMYC2 (AtbHLH006) is a transcriptional activator in ABA-inducible gene expression under drought stress in plants as it regulates the expression of *rd22* [21]. The *rd22* gene is a dehydration-responsive gene that is activated in *Arabidopsis* plants by exogenous ABA [122]. *AtbHLH122* responds to drought stress via an ABA-independent pathway as it represses CYP707A3, an important ABA 8′-hydroxylase [123], increasing ABA content, and then expressing ABA-inducible genes. It functions as a positive regulator in drought, salt and osmotic signaling [50]. OsbHLH148 also responds to drought stress via a jasmonate signaling pathway. Jasmonates (JAs) are a class of oxygen-containing lipid derivatives (oxylipins) that are considered plant hormones necessary for plant growth and environmental adaptation [124]. In the absence of stress and JA, OsbHLH148 is repressed through its interaction with OsJAZ. Upon exposure to drought stress, JA increases, leading to the degradation of OsJAZ proteins. The released OsbHLH148 activates drought tolerance genes, including *OsDREB1s*, leading to drought tolerance [51] (Figure 4A).

**Figure 5:**
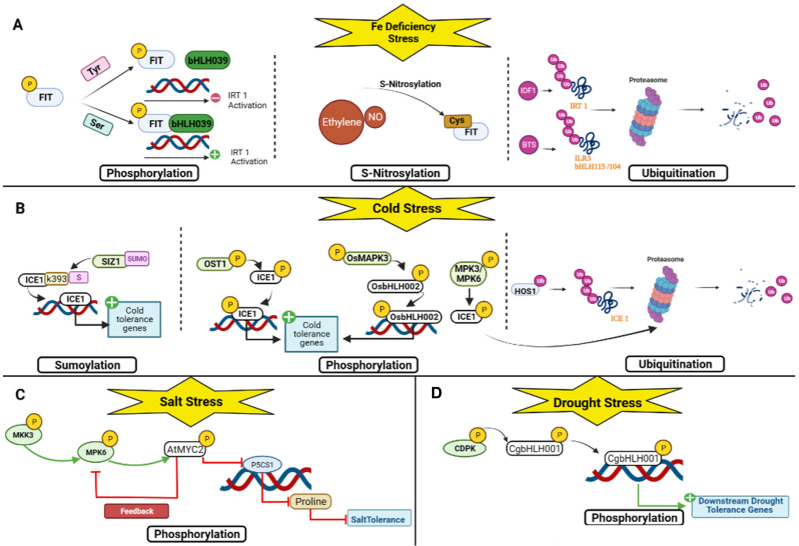
Post-translational regulations of bHLH TF under abiotic stresses. (**A**) For drought stress; (**B**) for salt stress; (**C**) for cold stress; and (**D**) for nutrient deficiency stress.

Under drought and salt stress, the overexpression of CgbHLH001 can confer stress tolerance. Regulated by phosphorylation by a protein kinase such as calcium-dependent protein kinase (CDPK), CgbHLH001 acts as a positive regulator in controlling downstream relevant genes, thereby reducing ROS production and enhancing ROS scavenging ability and improving physiological performance [18] (Figure 5D).

## 5. Salt Stress Response

Salinity has two main effects on plants. It either induces osmotic stress or induces the instability of ions. Several bHLH transcription factors have been characterized in rice. There is *OrbHLH001*, a homologue of *ICE1* (*AtbHLH116*), which activates *OsAKT1*, which enhances Na+ efflux and K+ uptake, thus controlling the Na+/K+ ratio in salt stress to confer salt stress tolerance [52]. BEAR1 (OsbHLH014), a bHLH transcription factor, plays a vital role in rice salt stress response. After receiving the salt stress signal, the BEAR1 protein as a transcriptional activator induces two pathways. On the one hand, it controls the gene expression level of the salt response signaling cascade. On the other hand, it regulates the Na+ /K+ balance and membrane stabilization genes and thus induces salt stress tolerance [53]. OsbHLH062 is involved in a jasmonate-dependent transcription regulatory module complex: Osbhlh062-JAZ9-OsNINJA. OsJAZ9 represents a transcriptional regulator and an essential element that modulates salt stress tolerance by binding OsbHLH062, a transcriptional activator, to OsNINJA, an essential repressor of basal jasmonate (JA) signaling. Under salt stress, JA levels increase. The JAZ protein OsJAZ9 is recruited by SCFCOI1 for ubiquitination by the 26S proteasome, leading to its degradation, releasing OsNINJA, and allowing OsbHLH062 to bind to the E-box and activate target genes. OsbHLH062 can bind to the E-box cis-element and the promoter of OsHAK21, including some of the ion transporters, thereby conferring salt stress tolerance [54]. *AtbHLH122* inhibits *CYP707A3* gene expression under NaCl stress [50]. In addition, Krishnamurthy et al. (2019) identified AtMYC2 and *AtbHLH122* as upstream regulators of ABA-mediated *AtNHX1* and *AtNHX6*, both Na+/H+ exchangers. In addition, the overexpression of EcbHLH57 enhanced tobacco resistance to salt stress by increasing the expression of stress-responsive genes such as *LEA14*, *rd29A*, *rd29B*, *SOD*, *APX*, *ADH1*, *HSP70*, and *PP2C* [55]. *BvbHLH93* regulates salt stress tolerance by enhancing antioxidant activity and reducing ROS production, thereby maintaining ion homeostasis, but it needs further investigation at the transcriptional level [56]. SlbHLHopt is an essential regulator of water deficit and NaCl stress in transgenic *Arabidopsis* since it increases flavonoid content [57]. In *Arabidopsis*, *AtbHLH112* regulates the expression of genes involved in abiotic stress tolerance by increasing proline levels and reducing ROS accumulation and water loss through the expression of *Proline Dehydrogenase 1* (*ProDH*) [58]. OsbHLH035 responds to salt stress through an ABA-independent pathway by indirectly mediating the expression of *OsHKT1*s such as *OsHKT1;3* and *OsHKT1;5*, which are sodium transporters that transfer Na+ from the xylem into xylem parenchyma cells, thus preventing the accumulation of Na+ in aerial tissues, since a higher concentration of Na+ in the roots causes osmotic stress [59]. *OrbHLH2* in transgenic *Arabidopsis* improved salt tolerance through an ABA-independent pathway by increasing expression levels of *DREB1A/CBF* to confer improved plant tolerance to salt stress; however, more research is needed to determine its mode of action [60]. *PgbHLH102* could respond to salt stress and higher salt concentrations, but its mode of action needs further investigation [61] (Figure 4B). Through an MKK3-MPK6-MYC2 cascade, AtMYC2 (AtbHLH006) also functions as a negative regulator of salt tolerance in *A. thaliana*. In brief, the MKK3-MPK6 module phosphorylates and hence activates AtMYC2 in response to salt stress. AtMYC2 binds to *P5CS1*, which is the main contributor to proline accumulation induced by stress [125], and regulates salt tolerance in *Arabidopsis thaliana* by inhibiting the synthesis of *P5CS1* and proline [126] (Figure 5C).

## 6. Cold Stress Response

Cold stress, including chilling, adversely affects plant growth and development, limits the geographical distribution of plant species, and reduces crop yields worldwide [127]. For acclimation to cold stress, cold tolerance requires a downstream cascade of transcriptional regulation of target genes. Cold-responsive genes contain DRE/CRT cis-elements with a core sequence CCGAC [25,128]. C-repeat Binding Factors (*CBFs*) (*CBF1*, *CBF2*, and *CBF3*; also known as *DREB1b*, *DREB1c*, and *DREB1a*, respectively) are known genes that bind to these elements, triggering transcription of cold-responsive genes [129]. Since *CBFs* are only induced after 15 min of cold exposure, the intervention of another TF present in the cell is required [130]. In *Arabidopsis*, the most well-defined pathway is the *ICE 1* (*AtbHLH116*) transcription factor, an ABA-independent pathway that binds to the CBF promoter and activates transcription of *CBF1/3*, which in turn induces the *CRT/BRE* genes responsible for cold tolerance [62]. *ICE2* (*BAC42644*), a homologue of *ICE1* (*AtbHLH116*), mediates the same ABA-independent pathway in cold tolerance [63]. *MdCIbHLH1* also interferes with the *CBF* pathway by activating the transcription of *CBF 1* and *2* [64]. *PuICE1* demonstrates a *CBF/DREB* pathway that requires cold-induced protein-protein interactions with the Hepta-helical protein 1 (PuHHP1), which acts as a positive regulator of ABA-mediated stomatal closure in response to cold stress, thus increasing the levels of PuDREBa transcripts and positively regulating cold stress; however, more research is needed to experimentally clarify the physiological mechanism between PuHHP1 and *PuICE1*. [65,131]. In rice, *OsbHLH1* expression is significant for cold tolerance as it might also regulate *CBF/DREB1* gene expression [66]. The IbbHLH79 protein, an *ICE1*-like gene, can activate the *CBF* pathway and IbbHLH79-overexpressing transgenic plants show enhanced cold tolerance [15]. Using the *NtCBFs* signaling pathways, cold-activated *NtbHLH123* and *ZjbHLH76/ZjICE1* regulate the expression of their target genes or regulate stress-responsive genes such as CBFs associated with ROS scavenging, resulting in improved tolerance to cold stress [67,68,69]. *VaICE1* and *VaICE2 ICE*-like TF play a positive role in freezing tolerance and influence cold stress-related factors such as electrolyte leakage and proline and malondialdehyde (MDA) levels, thereby reducing ROS damage and improving osmotic protection [70]. The overexpression of *DlICE1* in transgenic *Arabidopsis thaliana* enhances cold tolerance by increasing proline content, reducing ion leakage and accumulation of MDA and ROS [71]. *CsbHLH18* mediates a cold response by regulating antioxidant genes by binding to and activating the *CsPOD* promoter, thereby inducing ROS scavenging [72]. *PtrbHLH* also responds to cold stress by modulating POD and CAT [73,74]. *RmICE1* also responds to cold stress by regulating antioxidant genes [75]. Several *PavbHLH* also demonstrated a cold response regulatory mechanism, but the molecular details require further studies [76] (Figure 4C). HOS1 encodes a ring finger protein with E3 ligase activity that reduces *ICE1* (*AtbHLH116*) activity via the ubiquitination/proteasome pathway [132,133]. In addition, OST1 phosphorylates *ICE1* (*AtbHLH116*) and inhibits HOS1-mediated degradation of *ICE1*, since the OST1 protein competes with HOS1 for binding to *ICE1*, thereby releasing *ICE1* from the HOS1–*ICE1* complex. The dual role of OST1 helps enhance *ICE1* stability to increase *CBF* expression and freeze tolerance [134]. MKK2 is also activated by cold stress in plants and consequently activates MPK4 and MPK6 [135]. In brief, MPK3/6-mediated phosphorylation promotes *ICE1* (*AtbHLH116*) degradation, with Ser94, Thr366, and Ser403 being important sites for phosphorylation-dependent degradation [28]. Subsequently, when *ICE1* (*AtbHLH116*) levels accumulate, it is phosphorylated by MPK3/MPK6 and consequently cleared via the 26S proteasome pathway, which inhibits *CBF*-dependent cold signaling [136]. Another positive regulation is found in SUMO conjugation, which enhances *ICE1* (*AtbHLH116*) activity. SIZ1 (SAP and Miz) encodes a SUMO-E3 ligase that is necessary for freezing tolerance, facilitates sumoylation of *ICE1* (*AtbHLH116*), and recognizes K393 as the SUMO conjugation residue in the protein. SIZ-mediated SUMO1 conjugation to K393 affects the activity of *ICE1* (*AtbHLH116*) to control *CBF3/DREB1A* expression and prevents access to the ubiquitination complex. A K393R mutation blocks *ICE1* (*AtbHLH116*) sumoylation, represses expression of *CBF3/DREB1A* and its regulon genes, and reduces cold tolerance [29]. Under cold stress, active OsMAPK3 phosphorylates OsbHLH002, accumulating phospho-OsbHLH002, promoting trehalose-6-phosphate phosphatase 1 (OsTPP1) expression, and increasing trehalose content and resistance to chilling damage [137] (Figure 5B).

## 7. Iron Deficiency Response

Iron (Fe) is an essential element for plant survival and development as it is involved in several vital processes: photosynthesis, DNA synthesis, respiration, and chlorophyll synthesis. In *Arabidopsis*, FIT (AtbHLH029) is a well-known TF that plays a crucial role in Fe uptake via the first strategy [77], which consists of the acidification of the external environment mediated by membrane proton pumps, the *Autoinhibited Plasma Membrane H+/ATPases* (*AHA2*) [138], to release and solubilize the iron. It is then reduced from ferric iron to ferrous iron by *Ferric Reduction Oxidase 2* (*FRO2*) [139] and transported through specific channels within the *Iron-Regulated Transporter 1* (*IRT1*) in the plant [140]. It binds to the fer uptake genes *FRO2* and *IRT1*. Since it cannot function alone, it forms heterodimers with the four bHLH TFs AtbHLH38, AtbHLH39, AtbHLH100, and AtbHLH101 [78,79,141,142]. There is also another protein, POPEYE (PYE) (AtbHLH47), that responds to Fe deficiency by using a network independent of FIT [80]. The dimers of ILR3 (AtbHLH105) and AtbHLH104 or ILR3 (AtbHLH105) and AtbHLH34 regulate PYE (AtbHLH47), which interacts with two bHLH transcription factors, ILR3 (AtbHLH105) and AtbHLH115 [81,82,143]. Four other bHLH partners of FIT (AtbHLH029) (AtbHLH18, AtbHLH19, AtbHLH20, and AtbHLH25) promote its degradation in response to JA induction, thereby antagonizing the activity of AtbHLH38, AtbHLH39, AtbHLH100, and AtbHLH101 and limiting Fe uptake [83]. At the transcriptional level, the active and inactive states are distinguished by specific covalent modifications. Suppose the transition from the inactive state to the active state is bottlenecked. In that case, this could be achieved by limiting the enzymes that can confer or remove covalent modifications to “activate” FIT (AtbHLH029). FIT (AtbHLH029) could be in a negative feedback loop, limiting its abundance. In the context of Fe regulation, the rapid switching off of FIT (AtbHLH029) could be to prevent Fe toxicity symptoms [39]. A study by Zhao et al. showed that *chrysanthemum* CmbHLH1 promoted iron absorption through H+-ATPase-mediated rhizosphere acidification. The second strategy is not based on the reduction of iron [84]. Nonetheless, it is chelated by phytosiderophores (PS) [144] and then transported by a specific transporter, the oligopeptide transporter YS1. This strategy led to the discovery of the transcription factors IDEF1 and IDEF2 in rice, which control the expression of phytosiderophore biosynthesis and YSL2 [145,146]. *IRO2* in rice is a close homologue of bHLH39 and positively regulates phytosiderophore biosynthesis and YSL15 [41]. In rice, the iron-related transcription factor 3 (*OsIRO3*) (*OsbHLH063*), which belongs to the bHLH gene family, plays a critical role in maintaining iron homeostasis in an iron-deficient environment, as it negatively controls transcript levels of *OsIRO2* (*OsbHLH056*) [85,86,87]. Ogo et al. (2007) found that the overexpression of *OsIRO2/OsbHLH056* promotes iron uptake in rice following the 2nd strategy; however, its mode of action still needs further studies. Wang et al. studied the role of a new rice bHLH-type transcription factor, OsbHLH156, in iron homeostasis and found that OsbHLH156 greatly increased iron deficiency. They concluded that OsbHLH156 is required for a strategy II uptake mechanism in rice (Figure 4D). Ethylene (ET) and Nitric Oxide (NO) are required for complete upregulation of Fe-deficiency gene expression and FIT (AtbHLH029) protein abundance as they increase the accumulation of FIT by inhibiting proteasomal FIT (AtbHLH029) and thereby prevent its degradation. Therefore, they stabilize FIT (AtbHLH029) expression by nitrosylation of Cys residues present in FIT [147]. Therefore, this finding requires further elucidation. To limit Fe accumulation in plants, a RING-type E3 ubiquitin ligase called Degradation Factor 1 (IDF1) (AT4G30370) is directly involved in the degradation of *IRT1* through ubiquitination [148,149]. To limit the excess of Fe, which can be toxic to plants, there is BRUTUS (BTS) (AT3G18290), a RING-type E3 ubiquitin ligase that plays a role as a negative regulator since it is induced in Fe deficiency and has been shown to target the bHLH transcription factors ILR3 (AtbHLH105) and bHLH115/104 for degradation via the 26S proteasome by its RING domain (E3 ligase), thus inhibiting the formation of heterodimer complexes of PYE/PYEL (AtbHLH47). This includes disrupting the activation of Fe-deficiency genes regulated by PYE (AtbHLH47). BTS (AT3G18290) contains a hemerythrin-like domain that can bind Fe. Fe binding to the hemerythrin-like domain of BTS is involved in its destabilization and subsequent degradation [27,150]. On the one hand, it has been shown that the phosphorylation of Ser 221 and Ser 272 in FIT (AtbHLH029) positively regulates FIT accumulation in the nucleus, enabling its dimerization to AtbHLH039 and its activation of *IRT1* promoters [39]. On the other hand, phosphorylation of Tyr238 and Tyr278 reduces and inhibits heterodimerization of FIT (AtbHLH029) with AtbHLH039 and expression of *IRT1* promoters, rendering FIT (AtbHLH029) inactive [41] (Figure 5A).

## 8. Phosphorus and Nitrogen Deprivation Stress Response

Phosphorus (P) and Nitrogen (N) are both indispensable macronutrients for plant growth and crop productivity. *TabHLH1* is sensitive to external Pi and N deficiency stress. It confers enhanced tolerance to both Pi and N deficiency through the transcriptional modulation of a panel of genes encoding *Phosphate Transporter* (*PT*), *Nitrate Transporter* (*NRT*), and antioxidant enzymes [88]. *NRI1*, previously named *Nitrogen Starvation-Induced Gene 17* (*NSG17*) (BAD44756.1), a basic Helix–Loop–Helix (bHLH) TF, represses the transcriptional activators of N starvation-induced genes to regulate N starvation-specific responses when sufficient N is supplied to *C. reinhardtii* [89]. *OsPTF1* (AAO73566), responsible for tolerance to Pi starvation in rice, controls the Pi transport system in plants. *OsPTF1* is an essential element involved in higher root growth and consequently higher uptake rates of Pi, and it is also involved in the efficient use of Pi in plants. Therefore, *OsPTF1* has potential in engineering plants with higher Pi utilization efficiency [90] (Figure 4D).

## 9. Conclusions and Future Perspectives

To withstand environmental stresses, plants have evolved interdependent regulatory pathways that allow them to respond and adapt to the environment in a timely manner. Abiotic stress conditions affect many aspects of plant physiology and cause widespread changes in cellular processes. Research into plant defense mechanisms after abiotic stress is of great importance for subsequent breeding. As one of the most abundant transcription factor families in eukaryotes, members of the bHLH family have complex structures, large numbers, and diverse functions. Many studies have shown that bHLHs can regulate plant resistance to a variety of abiotic stresses. This review summarized the roles of the bHLH transcription factor family in the corresponding abiotic stresses in plants from two aspects. First, bHLH transcription factors show evolutionary differences in the conservative domain. Previous studies have shown that members of the same subfamily may regulate the synthesis of the same plant hormones [151]. Thus, there is reasonable speculation that members of the same subfamily may have evolved through genetic replication from the same ancestor. However, these assumptions still need to be verified. Second, we discussed how the bHLH transcription factor specifically regulates plant tolerance to abiotic stresses through transcriptional and post-translational modification pathways. Furthermore, with the development of transcriptome sequencing, transcriptome technology has been widely used in the study of plant stress tolerance. RNA-seq can quickly screen important functional genes by looking for genes with significant differences through samples, in order to find key nodes or genes for stress resistance at the transcriptional level for subsequent research. This naturally raises an interesting question: can bHLHs regulate plant tolerance to stress by controlling the synthesis of plant hormones or compounds via transcriptional or post-translational modification pathways?

## Figures and Tables

**Table 1:** bHLH transcription factors involved in plant response to abiotic stress.

Original Plant	Nomenclature	Stress Response	Regulation Type	Refs.
*Ipomoea batatas* (L.) *Lam.*	IbbHLH79	Cold	Positive regulation	[15]
*Arabidopsis thaliana* L.	rd22BP1/AtMYC2/	Drought	Positive regulation	[21]
	AtbHLH006			
*Arachis hypogaea* L.	AhbHLH112	Drought/salt	Positive regulation	[42]
*Oryza sativa*	OsWIH2/OsbHLH130	Drought	Positive regulation	[43]
*Fagopyrum tataricum*	FtbHLH3	Drought/oxidative	Unknown	[44]
*Solanum lycopersicum*	SlbHLH22	Drought	Positive regulation	[45]
*Zea mays* L.	ZmPIF1	Drought	Positive regulation	[46]
*Malus × domestica Borkh.*	MdCIB1	Drought	Positive regulation	[47]
*Arabidopsis thaliana* L.	AtbHLH68	Drought	Unknown	[48]
*Populus euphratica*	PebHLH35	Drought	Positive regulation	[49]
*Arabidopsis thaliana* L.	AtbHLH122	Drought/salt	Positive regulation	[50]
*Oryza sativa*	OsbHLH148	Drought	Positive regulation	[51]
*Oriza rufipogon*	OrbHLH001	Salt/cold	Positive regulation	[52]
*Oryza sativa*	BEAR1/OsbHLH014	Salt	Positive regulation	[53]
*Oryza sativa*	OsbHLH062	Salt	Unknown	[54]
*Eleusine coracana* L.	EcbHLH57	Salt/oxidative/drought	Positive regulation	[55]
*Beta vulgaris* L.	BvbHLH93	Salt	Positive regulation	[56]
*Selaginella lepidophylla*	SlbHLHopt	Salt	Positive regulation	[57]
*Arabidopsis thaliana* L.	AtbHLH112	Drought/salt	Positive regulation	[58]
*Oryza sativa*	OsbHLH035	Salt	Unknown	[59]
*Oriza rufipogon*	OrbHLH2	Salt/osmotic	Positive regulation	[60]
*Panax ginseng* C.A. Meyer	PgbHLH102	Salt	Unknown	[61]
*Arabidopsis thaliana* L.	AtICE1/AtbHLH116	Cold	Positive regulation	[62]
*Arabidopsis thaliana* L.	ICE2	Cold	Positive regulation	[63]
*Malus × domestica Borkh.*	MdCIbHLH1	Cold	Positive regulation	[64]
*Pyrus ussuriensis*	PuICE1	Cold	Positive regulation	[65]
*Oryza sativa*	OsbHLH1	Cold	Positive regulation	[66]
*Nicotiana tabacum* L.	NtbHLH123	Cold/salt	Positive regulation	[67]
*Zoysia japonica*	ZjbHLH76/ZjICE1	Cold	Positive regulation	[68,69]
*Vitis amurensis*	VaICE1/VaICE2	Cold	Positive regulation	[70]
*Dimocarpus longan* Lour.	DlICE1	Cold	Positive regulation	[71]
*Citrus sinensis*	CsbHLH18	Cold/salt	Positive regulation	[72]
*Poncirus trifoliate*	PtrbHLH	Cold/oxidative	Positive regulation	[73,74]
*Rosa multiflora*	RmICE1	Cold/salt	Positive regulation	[75]
*Prunus avium* L.	PavbHLHs	Cold	Unknown	[76]
*Arabidopsis thaliana* L.	FIT/AtbHLH29	Fe deficiency	Unknown	[77]
*Arabidopsis thaliana* L.	AtbHLH38	Fe deficiency	Positive regulation	[78]
*Arabidopsis thaliana* L.	AtbHLH39	Fe deficiency	Positive regulation	[78]
*Arabidopsis thaliana* L.	AtbHLH100	Fe deficiency	Positive regulation	[79]
*Arabidopsis thaliana* L.	AtbHLH101	Fe deficiency	Positive regulation	[79]
*Arabidopsis thaliana* L.	PYE/AtbHLH47	Fe deficiency	Unknown	[80]
*Arabidopsis thaliana* L.	ILR3/AtbHLH105	Fe deficiency	Positive regulation/Negative regulation	[81]
*Arabidopsis thaliana* L.	AtbHLH104	Fe deficiency	Positive regulation	[82]
*Arabidopsis thaliana* L.	AtbHLH18	Fe deficiency	Negative regulation	[83]
*Arabidopsis thaliana* L.	AtbHLH19	Fe deficiency	Negative regulation	[83]
*Arabidopsis thaliana* L.	AtbHLH20	Fe deficiency	Negative regulation	[83]
*Arabidopsis thaliana* L.	AtbHLH25	Fe deficiency	Negative regulation	[83]
*Chrysanthemum morifolium*	CmbHLH1	Fe deficiency	Unknown	[84]
*Oryza sativa*	OsIRO3/OsbHLH2	Fe deficiency	Positive regulation	[85]
*Oryza sativa*	OsIRO2/OsbHLH056	Fe deficiency	Positive regulation	[86]
*Oryza sativa*	OsbHLH156	Fe deficiency	Positive regulation	[87]
*Triticum aestivum* L.	TabHLH1	Pi and N deficiency	Positive regulation	[88]
*Chlamydomonas reinhardtii*	NRI1	N starvation	Positive regulation	[89]
*Oryza sativa*	OsPTF1	Pi starvation	Positive regulation	[90]
*Nicotiana tabacum* L.	NtbHLH1	Fe deficiency	Positive regulation	[91]
*Zea mays* L.	ZmICE1	Cold	Positive regulation	[92]
*Oryza sativa*	OsPRI2/OsbHLH058	Fe deficiency	Positive regulation	[93]
	OsPRI3/OsbHLH059			
*Solanum lycopersicum*	SlICE1	Cold/salinity	Positive regulation	[94]
*Cucumis sativus* L.	CsbHLH041	Salt	Positive regulation	[95]
*Nicotiana tabacum* L.	NtbHLH86	Drought	Positive regulation	[96]
*Malus × domestica Borkh.*	MdbHLH3	Cold	Positive regulation	[97]
*Malus × domestica Borkh.*	MdbHLH106L	Salt	Positive regulation	[98]
*Malus × domestica Borkh.*	MdbHLH130	Salt	Positive regulation	[99]
*Pyrus ussuriensis*	PubHLH1	Cold	Positive regulation	[100]
*Arabidopsis thaliana* L.	AtbHLH92	Salt/osmotic stress	Unknown	[101]
*Arabidopsis thaliana* L.	AtAIB/AtbHLH17	Drought/salt	Positive regulation	[102]
*Fagopyrum tataricum*	FtbHLH2	Cold	Positive regulation	[103]
*Solanum tuberosum*	StbHLH45	Drought	Unknown	[104]
*Eucalyptus camaldulensis*	EcaICE1	Cold	Positive regulation	[105]
*Triticum aestivum* L.	TabHLH39	Osmotic	Unknown	[106]
*Arabidopsis thaliana* L.	AtNIG1/AtbHLH028	Salt	Positive regulation	[107]
*Glycine Max* (L.) *Merrill*	GmbHLH57	Fe deficiency	Unknown	[108]
*Glycine Max* (L.) *Merrill*	GmbHLH300	Fe deficiency	Unknown	[108]
*Zea mays* L.	ZmPIF3	Drought	Positive regulation	[109]
*Oryza sativa*	OsbHLH133	Fe deficiency	Negative regulation	[110]
*Oryza sativa*	OsPRI1/OsbHLH115	Fe deficiency	Positive regulation	[111]
*Arabidopsis thaliana L.*	AtbHLH121	Fe deficiency	Unknown	[112]
*Arabidopsis thaliana* L.	AtbHLH106	Salt	Positive regulation/Negative regulation	[113]
*Arabidopsis thaliana* L.	AtbHLH11	Fe deficiency	Negative regulation	[114]
*Oryza sativa*	OsbHLH006	Drought	Unknown	[115]
*Oryza sativa*	OsbHLH068	Salt	Positive regulation	[116]
*Vitis vinifera*	VvbHLH1	Drought/salt/cold	Positive regulation	[117]

## Data Availability

Not applicable.

## Abbreviations

ACT domain, small regulatory domains initially found in AK, chorismate mutase, and TyrA (prephenate dehydrogenase); TFs, transcription factors; ACE, ACTIVATOR FOR CELL ELONGATION; AIF, ATBS1 (ACTIVATION-TAGGED BRI1 SUPPRESSOR 1)-INTERACTING FACTOR; ARF, AUXIN RESPONSE FACTOR; BEE, BR ENHANCED EXPRESSION; BEH, BES1-HOMOLOGUE; BES1, BRI1 EMS-SUPRESSOR 1; bHLH, basic helix–loop–helix; BIM, BES1-INTERACTING MYC-LIKE; BR, brassinosteroid; BRZ, brassinazole; CES, CESTA; CIB, CRYPTOCHROME INTERACTING bHLH; CIL, CIB1-LIKE PROTEIN; HOS1, high expression of osmotically responsive gene 1; NCED, 9-cis-epoxycarotenoid dioxygenase; CBF4, C-repeat-binding factor; GA, gibberellin; HBI1, HOMOLOGUE OF BEE2 INTERACTING WITH IBH1; HFR1, LONG HYPOCOTYL IN FAR-RED 1; IBH1, ILI1 BINDING BHLH 1; IBL1, IBH1-LIKE 1; KDR, KIDARI; PAR, PHYTOCHROME RAPIDLY REGULATED; phy, phytochrome; PIF, PHYTOCHROME INTERACTING FACTOR; PIL1, PIF3-LIKE 1; PRE, PACLOBUTRAZOL RESISTANCE; YSL, an iron-phytosiderophore transporter; ABA, abscisic acid; JA, jasmonic acid; P5CS1, pyrroline-5-carboxylate synthetase 1; FIT, FER-LIKE IRON DEFICIENCY-INDUCED TRANSCRIPTION FACTOR.

## References

[1] Pires N., Dolan L. (2010). Origin and Diversification of Basic-Helix-Loop-Helix Proteins in Plants. Mol. Biol. Evol..

[2] Massari M.E., Murre C. (2000). Helix-Loop-Helix Proteins: Regulators of Transcription in Eucaryotic Organisms. Mol. Cell. Biol..

[3] Heim M.A., Jakoby M., Werber M., Martin C., Weisshaar B., Bailey P.C. (2003). The Basic Helix-Loop-Helix Transcription Factor Family in Plants: A Genome-Wide Study of Protein Structure and Functional Diversity. Mol. Biol. Evol..

[4] Li X., Duan X., Jiang H., Sun Y., Tang Y., Yuan Z., Guo J., Liang W., Chen L., Yin J. (2006). Genome-Wide Analysis of Basic/Helix-Loop-Helix Transcription Factor Family in Rice and Arabidopsis. Plant Physiol..

[5] Murre C., McCaw P.S., Baltimore D. (1989). A new DNA binding and dimerization motif in immunoglobulin enhancer binding, daughterless, MyoD, and myc proteins. Cell.

[6] Ledent V., Vervoort M. (2001). The Basic Helix-Loop-Helix Protein Family: Comparative Genomics and Phylogenetic Analysis. Genome Res..

[7] Atchley W.R., Fitch W.M. (1997). A natural classification of the basic helix–loop–helix class of transcription factors. Proc. Natl. Acad. Sci. USA.

[8] Carretero-Paulet L., Galstyan A., Roig-Villanova I., Martínez-García J.F., Bilbao-Castro J.R., Robertson D.L. (2010). Genome-wide classification and evolutionary analysis of the bHLH family of transcription factors in Arabidopsis, poplar, rice, moss, and algae. Plant Physiol..

[9] Bailey P.C., Martin C., Toledo-Ortiz G., Quail P.H., Huq E., Heim M.A., Jakoby M., Werber M., Weisshaar B. (2003). Update on the Basic Helix-Loop-Helix Transcription Factor Gene Family in *Arabidopsis thaliana*. Plant Cell.

[10] Zhao K., Li S., Yao W., Zhou B., Li R., Jiang T. (2018). Characterization of the basic helix–loop–helix gene family and its tissue-differential expression in response to salt stress in poplar. PeerJ.

[11] Wu Y., Wu S., Wang X., Mao T., Bao M., Zhang J. (2022). Genome-wide identification and characterization of the bHLH gene family in an ornamental woody plant Prunus mume. Hortic. Plant J..

[12] Zhao W., Liu Y., Li L., Meng H., Yang Y., Dong Z., Wang L., Wu G. (2021). Genome-Wide Identification and Characterization of bHLH Transcription Factors Related to Anthocyanin Biosynthesis in Red Walnut (*Juglans regia* L.). Front. Genet..

[13] Salih H., Tan L., Htet N.N.W. (2021). Genome-Wide Identification, Characterization of bHLH Transcription Factors in Mango. Trop. Plant Biol..

[14] Ali A., Javed T., Zaheer U., Zhou J.-R., Huang M.-T., Fu H.-Y., Gao S.-J. (2021). Genome-Wide Identification and Expression Profiling of the bHLH Transcription Factor Gene Family in Saccharum spontaneum Under Bacterial Pathogen Stimuli. Trop. Plant Biol..

[15] Jin R., Kim H.S., Yu T., Zhang A., Yang Y., Liu M., Yu W., Zhao P., Zhang Q., Cao Q. (2021). Identification and function analysis of bHLH genes in response to cold stress in sweetpotato. Plant Physiol. Biochem..

[16] Tan C., Qiao H., Ma M., Wang X., Tian Y., Bai S., Hasi A. (2021). Genome-Wide Identification and Characterization of Melon bHLH Tran-scription Factors in Regulation of Fruit Development. Plants.

[17] Liu R., Song J., Liu S., Chen C., Zhang S., Wang J., Xiao Y., Cao B., Lei J., Zhu Z. (2021). Genome-wide identification of the Capsicum bHLH transcription factor family: Discovery of a candidate regulator involved in the regulation of species-specific bioactive metabolites. BMC Plant Biol..

[18] Zhou X., Liao Y., Kim S.-U., Chen Z., Nie G., Cheng S., Ye J., Xu F. (2020). Genome-wide identification and characterization of *bHLH* family genes from *Ginkgo biloba*. Sci. Rep..

[19] Castelain M., Le Hir R., Bellini C. (2012). The non-DNA-binding bHLH transcription factor PRE3/bHLH135/ATBS1/TMO7 is involved in the regulation of light signaling pathway in Arabidopsis. Physiol. Plant..

[20] Qi Y., Zhou L., Han L., Zou H., Miao K., Wang Y. (2020). *PsbHLH1*, a novel transcription factor involved in regulating anthocyanin biosynthesis in tree peony (*Paeonia suffruticosa*). Plant Physiol. Biochem..

[21] Abe H., Urao T., Ito T., Seki M., Shinozaki K., Yamaguchi-Shinozaki K. (2002). Arabidopsis AtMYC2 (bHLH) and AtMYB2 (MYB) Function as Transcriptional Activators in Abscisic Acid Signaling. Plant Cell.

[22] Zhang L.Y., Bai M.Y., Wu J., Zhu J.Y., Wang H., Zhang Z., Wang W., Sun Y., Zhao J., Sun X. (2009). Antagonistic HLH/bHLH transcription factors mediate brassino-steroid regulation of cell elongation and plant development in rice and Arabidopsis. Plant Cell.

[23] Bernhardt C., Lee M.M., Gonzalez A., Zhang F., Lloyd A., Schiefelbein J. (2003). Faculty Opinions recommendation of the bHLH genes GLABRA3 (GL3) and ENHANCER OF GLABRA3 (EGL3) specify epidermal cell fate in the Arabidopsis root. Development.

[24] Morohashi K., Zhao M., Yang M., Read B., Lloyd A., Lamb R., Grotewold E. (2007). Participation of the Arabidopsis bHLH Factor GL3 in Trichome Initiation Regulatory Events. Plant Physiol..

[25] Yamaguchi-Shinozaki K., Shinozaki K. (1994). A novel cis-acting element in an Arabidopsis gene is involved in responsiveness to drought, low-temperature, or high-salt stress. Plant Cell.

[26] Krishnamurthy P., Vishal B., Khoo K., Rajappa S., Loh C.-S., Kumar P.P. (2019). Expression of AoNHX1 increases salt tolerance of rice and Arabidopsis, and bHLH transcription factors regulate AtNHX1 and AtNHX6 in Arabidopsis. Plant Cell Rep..

[27] Selote D., Samira R., Matthiadis A., Gillikin J.W., Long T.A. (2015). Iron-binding e3 ligase mediates iron response in plants by targeting basic helix-loop-helix transcription factors. Plant Physiol..

[28] Zhao C., Wang P., Si T., Hsu C.-C., Wang L., Zayed O., Yu Z., Zhu Y., Dong J., Tao W.A. (2017). MAP Kinase Cascades Regulate the Cold Response by Modulating ICE1 Protein Stability. Dev. Cell.

[29] Miura K., Jin J.B., Lee J., Yoo C.Y., Stirm V., Miura T., Ashworth E.N., Bressan R.A., Yun D.J., Hasegawa P.M. (2007). SIZ1-mediated sumoylation of ICE1 controls CBF3/DREB1A ex-pression and freezing tolerance in Arabidopsis. Plant Cell..

[30] Ferre-D’Amare A.R., Prendergast G.C., Ziff E.B., Burley S.K. (1993). Recognition by Max of its cognate DNA through a dimeric b/HLH/Z domain. Nature.

[31] Toledo-Ortiz G., Huq E., Quail P.H. (2003). The Arabidopsis Basic/Helix-Loop-Helix Transcription Factor Family. Plant Cell.

[32] Stevens J.D., Roalson E., Skinner M.K. (2008). Phylogenetic and expression analysis of the basic helix-loop-helix transcription factor gene family: Genomic approach to cellular differentiation. Differentiation.

[33] Jones S. (2004). An overview of the basic helix-loop-helix proteins. Genome Biol..

[34] Yin Y., Vafeados D., Tao Y., Yoshida S., Asami T., Chory J. (2005). A new class of transcription factors mediates brassinosteroid-regulated gene expression in Arabidopsis. Cell.

[35] Khanna R., Huq E., Kikis E.A., Al-Sady B., Lanzatella C., Quail P.H. (2004). A Novel Molecular Recognition Motif Necessary for Targeting Photoactivated Phytochrome Signaling to Specific Basic Helix-Loop-Helix Transcription Factors. Plant Cell.

[36] Verma S., Nizam S., Verma P.K. (2013). Biotic and Abiotic Stress Signaling in Plants. Stress Signal. Plants Genom. Proteom. Perspect..

[37] Suzuki N., Rivero R.M., Shulaev V., Blumwald E., Mittler R. (2014). Abiotic and biotic stress combinations. New Phytol..

[38] Wu H., Ye H., Yao R., Zhang T., Xiong L. (2015). OsJAZ9 acts as a transcriptional regulator in jasmonate signaling and modulates salt stress tolerance in rice. Plant Sci..

[39] Gratz R., Manishankar P., Ivanov R., Köster P., Mohr I., Trofimov K., Steinhorst L., Meiser J., Mai H.-J., Drerup M. (2019). CIPK11-Dependent Phosphorylation Modulates FIT Activity to Promote Arabidopsis Iron Acquisition in Response to Calcium Signaling. Dev. Cell.

[40] Qiu J.R., Huang Z., Xiang X.Y., Xu W.X., Wang J.T., Chen J., Song L., Xiao Y., Li X., Ma J. (2020). MfbHLH38, a Myrothamnus flabellifolia bHLH transcription factor, confers tolerance to drought and salinity stresses in Arabidopsis. BMC Plant Biol..

[41] Wang S., Li L., Ying Y., Wang J., Shao J.F., Yamaji N., Whelan J., Ma J.F., Shou H. (2019). A transcription factor OsbHLH156 regulates Strategy II iron acquisition through localising IRO2 to the nucleus in rice. New Phytol..

[42] Li C., Yan C., Sun Q., Wang J., Yuan C., Mou Y., Shan S., Zhao X. (2021). The bHLH transcription factor *AhbHLH112* improves the drought tolerance of peanut. BMC Plant Biol..

[43] Gu X., Gao S., Li J., Song P., Zhang Q., Guo J., Wang X., Han X., Wang X., Zhu Y. (2021). The bHLH transcription factor regulated gene OsWIH2 is a positive regulator of drought tolerance in rice. Plant Physiol. Biochem..

[44] Yao P.-F., Li C.-L., Zhao X.-R., Li M.-F., Zhao H.-X., Guo J.-Y., Cai Y., Chen H., Wu Q. (2017). Overexpression of a Tartary Buckwheat Gene, FtbHLH3, Enhances Drought/Oxidative Stress Tolerance in Transgenic Arabidopsis. Front. Plant Sci..

[45] Waseem M., Rong X., Li Z. (2019). Dissecting the Role of a Basic Helix-Loop-Helix Transcription Factor, SlbHLH22, Under Salt and Drought Stresses in Transgenic *Solanum lycopersicum* L.. Front. Plant Sci..

[46] Gao Y., Wu M., Zhang M., Jiang W., Ren X., Liang E., Zhang D., Zhang C., Xiao N., Li Y. (2018). A maize phytochrome-interacting factors protein ZmPIF1 enhances drought tolerance by inducing stomatal closure and improves grain yield in *Oryza sativa*. Plant Biotechnol. J..

[47] Ren Y.-R., Yang Y.-Y., Zhao Q., Zhang T.-E., Wang C.-K., Hao Y.-J., You C.-X. (2021). MdCIB1, an apple bHLH transcription factor, plays a positive regulator in response to drought stress. Environ. Exp. Bot..

[48] Le Hir R., Castelain M., Chakraborti D., Moritz T., Dinant S., Bellini C. (2017). At*bHLH68* transcription factor contributes to the regulation of ABA homeostasis and drought stress tolerance in *Arabidopsis thaliana*. Physiol. Plant..

[49] Dong Y., Wang C., Han X., Tang S., Liu S., Xia X., Yin W. (2014). A novel bHLH transcription factor PebHLH35 from Populus euphratica confers drought tolerance through regulating stomatal development, photosynthesis and growth in *Arabidopsis*. Biochem. Biophys. Res. Commun..

[50] Liu W., Tai H., Li S., Gao W., Zhao M., Xie C., Li W.X. (2014). bHLH122 is important for drought and osmotic stress resistance in Ara-bidopsis and in the repression of ABA catabolism. New Phytol..

[51] Seo J.-S., Joo J., Kim M.-J., Kim Y.-K., Nahm B.H., Song S.I., Cheong J.-J., Lee J.S., Kim J.-K., Choi Y.D. (2011). OsbHLH148, a basic helix-loop-helix protein, interacts with OsJAZ proteins in a jasmonate signaling pathway leading to drought tolerance in rice. Plant J..

[52] Chen Y., Li F., Ma Y., Chong K., Xu Y. (2013). Overexpression of OrbHLH001, a putative helix–loop–helix transcription factor, causes increased expression of AKT1 and maintains ionic balance under salt stress in rice. J. Plant Physiol..

[53] Teng Y., Lv M., Zhang X., Cai M., Chen T. (2022). BEAR1, a bHLH Transcription Factor, Controls Salt Response Genes to Regulate Rice Salt Response. J. Plant Biol..

[54] Singh A.P., Pandey B.K., Mehra P., Heitz T., Giri J. (2020). OsJAZ9 overexpression modulates jasmonic acid biosynthesis and potassium deficiency responses in rice. Plant Mol. Biol..

[55] Babitha K.C., Vemanna R.S., Nataraja K.N., Udayakumar M. (2015). Overexpression of EcbHLH57 Transcription Factor from Eleusine coracana L. in Tobacco Confers Tolerance to Salt, Oxidative and Drought Stress. PLoS ONE.

[56] Wang Y., Wang S., Tian Y., Wang Q., Chen S., Li H., Ma C., Li H. (2021). Functional Characterization of a Sugar Beet *BvbHLH93* Transcription Factor in Salt Stress Tolerance. Int. J. Mol. Sci..

[57] Ariyarathne M.A., Wone B.W. (2022). Overexpression of the Selaginella lepidophylla bHLH transcription factor enhances water-use efficiency, growth, and development in Arabidopsis. Plant Sci..

[58] Liu Y., Ji X., Nie X., Qu M., Zheng L., Tan Z., Zhao H., Huo L., Liu S., Zhang B. (2015). Arabidopsis AtbHLH112 regulates the expression of genes involved in abiotic stress tolerance by binding to their E-box and GCG-box motifs. New Phytol..

[59] Chen H.-C., Cheng W.-H., Hong C.-Y., Chang Y.-S., Chang M.-C. (2018). The transcription factor OsbHLH035 mediates seed germination and enables seedling recovery from salt stress through ABA-dependent and ABA-independent pathways, respectively. Rice.

[60] Zhou J., Li F., Wang J.-L., Ma Y., Chong K., Xu Y.-Y. (2009). Basic helix-loop-helix transcription factor from wild rice (OrbHLH2) improves tolerance to salt- and osmotic stress in *Arabidopsis*. J. Plant Physiol..

[61] Zhu L., Zhao M., Chen M., Li L., Jiang Y., Liu S., Jiang Y., Wang K., Wang Y., Sun C. (2020). The bHLH gene family and its response to saline stress in Jilin ginseng, Panax ginseng C.A. Meyer. Mol. Genet. Genom..

[62] Chinnusamy V., Ohta M., Kanrar S., Lee B.-H., Hong X., Agarwal M., Zhu J.-K. (2003). ICE1: A regulator of cold-induced transcriptome and freezing tolerance in *Arabidopsis*. Genes Dev..

[63] Fursova O.V., Pogorelko G.V., Tarasov V.A. (2009). Identification of ICE2, a gene involved in cold acclimation which determines freezing tolerance in Arabidopsis thaliana. Gene.

[64] Feng X.-M., Zhao Q., Zhao L.-L., Qiao Y., Xie X.-B., Li H.-F., Yao Y.-X., You C.-X., Hao Y.-J. (2012). The cold-induced basic helix-loop-helix transcription factor gene MdCIbHLH1 encodes an ICE-like protein in apple. BMC Plant Biol..

[65] Huang X., Li K., Jin C., Zhang S. (2015). ICE1 of Pyrus ussuriensis functions in cold tolerance by enhancing PuDREBa transcriptional levels through interacting with PuHHP1. Sci. Rep..

[66] Wang Y.-J., Zhang Z.-G., He X.-J., Zhou H.-L., Wen Y.-X., Dai J.-X., Zhang J.-S., Chen S.-Y. (2003). A rice transcription factor OsbHLH1 is involved in cold stress response. Theor. Appl. Genet..

[67] Zhao Q., Xiang X., Liu D., Yang A., Wang Y. (2018). Tobacco transcription factor NtbHLH123 confers tolerance to cold stress by regulating the NtCBF pathway and reactive oxygen species homeostasis. Front. Plant Sci..

[68] Zuo Z.F., Sun H.J., Lee H.Y., Kang H.G. (2021). Identification of bHLH genes through genome-wide association study and antisense expression of ZjbHLH076/ZjICE1 influence tolerance to low temperature and salinity in Zoysia japonica. Plant Sci..

[69] Zuo Z.-F., Kang H.-G., Park M.-Y., Jeong H., Sun H.-J., Song P.-S., Lee H.-Y. (2019). Zoysia japonica MYC type transcription factor ZjICE1 regulates cold tolerance in transgenic Arabidopsis. Plant Sci..

[70] Xu W., Jiao Y., Li R., Zhang N., Xiao D., Ding X., Wang Z. (2014). Chinese Wild-Growing Vitis amurensis ICE1 and ICE2 Encode MYC-Type bHLH Transcription Activators that Regulate Cold Tolerance in Arabidopsis. PLoS ONE.

[71] Yang X., Wang R., Hu Q., Li S., Mao X., Jing H., Zhao J., Hu G., Fu J., Liu C. (2019). DlICE1, a stress-responsive gene from Dimocarpus longan, enhances cold tolerance in transgenic Arabidopsis. Plant Physiol. Biochem..

[72] Geng J., Liu J.-H. (2018). The transcription factor CsbHLH18 of sweet orange functions in modulation of cold tolerance and homeostasis of reactive oxygen species by regulating the antioxidant gene. J. Exp. Bot..

[73] Huang X.S., Wang W., Zhang Q., Liu J.H. (2013). A basic helix-loop-helix transcription factor, PtrbHLH, of Poncirus trifoliata confers cold tolerance and modulates peroxidase-mediated scavenging of Hydrogen Peroxide. Plant Physiol..

[74] Geng J., Wei T.-L., Wang Y., Huang X., Liu J.-H. (2019). Overexpression of PtrbHLH, a basic helix-loop-helix transcription factor from Poncirus trifoliata, confers enhanced cold tolerance in pummelo (Citrus grandis) by modulation of H_2_O_2_ level via regulating a CAT gene. Tree Physiol..

[75] Luo P., Li Z., Chen W., Xing W., Yang J., Cui Y. (2020). Overexpression of RmICE1, a bHLH transcription factor from Rosa multiflora, enhances cold tolerance via modulating ROS levels and activating the expression of stress-responsive genes. Environ. Exp. Bot..

[76] Shen T., Wen X., Wen Z., Qiu Z., Hou Q., Li Z., Mei L., Yu H., Qiao G. (2021). Genome-wide identification and expression analysis of bHLH transcription factor family in response to cold stress in sweet cherry (*Prunus avium* L.). Sci. Hortic..

[77] Colangelo E.P., Guerinot M.L. (2004). The essential basic helix-loop-helix protein FIT1 is required for the iron deficiency response. Plant Cell.

[78] Yuan Y., Wu H., Wang N., Li J., Zhao W., Du J., Wang D., Ling H.-Q. (2008). FIT interacts with AtbHLH38 and AtbHLH39 in regulating iron uptake gene expression for iron homeostasis in Arabidopsis. Cell Res..

[79] Sivitz A.B., Hermand V., Curie C., Vert G. (2012). Arabidopsis bHLH100 and bHLH101 Control Iron Homeostasis via a FIT-Independent Pathway. PLoS ONE.

[80] Long T.A., Tsukagoshi H., Busch W., Lahner B., Salt D.E., Benfey P.N. (2010). The bHLH Transcription Factor POPEYE Regulates Response to Iron Deficiency in *Arabidopsis* Roots. Plant Cell.

[81] Samira R., Li B., Kliebenstein D., Li C., Davis E., Gillikin J.W., Long T.A. (2018). The bHLH transcription factor ILR3 modulates multiple stress responses in Arabidopsis. Plant Mol. Biol..

[82] Li X., Zhang H., Ai Q., Liang G., Yu D. (2016). Two bHLH Transcription Factors, bHLH34 and bHLH104, Regulate Iron Homeostasis in *Arabidopsis thaliana*. Plant Physiol..

[83] Cui Y., Chen C.-L., Cui M., Zhou W.-J., Wu H.-L., Ling H.-Q. (2018). Four IVa bHLH Transcription Factors Are Novel Interactors of FIT and Mediate JA Inhibition of Iron Uptake in *Arabidopsis*. Mol. Plant.

[84] Zhao M., Song A., Li P., Chen S., Jiang J., Chen F. (2014). A bHLH transcription factor regulates iron intake under Fe deficiency in chrysanthemum. Sci. Rep..

[85] Wang W., Ye J., Ma Y., Wang T., Shou H., Zheng L. (2020). OsIRO3 Plays an Essential Role in Iron Deficiency Responses and Regulates Iron Homeostasis in Rice. Plants.

[86] Ogo Y., Itai R.N., Nakanishi H., Kobayashi T., Takahashi M., Mori S., Nishizawa N.K. (2007). The rice bHLH protein OsIRO2 is an essential regulator of the genes involved in Fe uptake under Fe-deficient conditions. Plant J..

[87] Liang G., Zhang H., Li Y., Pu M., Yang Y., Li C., Lu C., Xu P., Yu D. (2020). *Oryza sativa* FER-LIKE FE DEFICIENCY-INDUCED TRANSCRIPTION FACTOR (OsFIT/OsbHLH156) interacts with OsIRO2 to regulate iron homeostasis. J. Integr. Plant Biol..

[88] Yang T., Hao L., Yao S., Zhao Y., Lu W., Xiao K. (2016). TabHLH1, a bHLH-type transcription factor gene in wheat, improves plant tolerance to Pi and N deprivation via regulation of nutrient transporter gene transcription and ROS homeostasis. Plant Physiol. Biochem..

[89] Jia M., Munz J., Lee J., Shelley N., Xiong Y., Joo S., Jin E., Lee J.H. (2022). The bHLH family NITROGEN-REPLETION INSENSITIVE1 represses nitrogen starvation-induced responses in Chlamydomonas reinhardtii. Plant J..

[90] Yi K., Wu Z., Zhou J., Du L., Guo L., Wu Y., Wu P. (2005). OsPTF1, a novel transcription factor involved in tolerance to phosphate starvation in rice. Plant Physiol..

[91] Li Y.Y., Sui X.Y., Yang J.S., Xiang X.H., Li Z.Q., Wang Y.Y., Zhou Z.C., Hu R.S., Liu D. (2020). A novel bHLH transcription factor, NtbHLH1, modulates iron homeostasis in tobacco (*Nicotiana tabacum* L.). Biochem. Biophys. Res. Commun..

[92] Lu X., Yang L., Yu M., Lai J., Wang C., McNeil D., Zhou M., Yang C. (2017). A novel Zea mays ssp. mexicana L. MYC-type ICE-like transcription factor gene ZmICE1, enhances freezing tolerance in transgenic Arabidopsis thaliana. Plant Physiol. Biochem..

[93] Zhang H., Li Y., Pu M., Xu P., Liang G., Yu D. (2020). *Oryza sativa* POSITIVE REGULATOR OF IRON DEFICIENCY RESPONSE 2 (OsPRI2) and OsPRI3 are involved in the maintenance of Fe homeostasis. Plant Cell Environ..

[94] Miura K., Shiba H., Ohta M., Kang S.W., Sato A., Yuasa T., Iwaya-Inoue M., Kamada H., Ezura H. (2012). SlICE1 encoding a MYC-type transcription factor controls cold tolerance in tomato, *Solanum lycopersicum*. Plant Biotechnol..

[95] Li J., Wang T., Han J., Ren Z. (2020). Genome-wide identification and characterization of cucumber bHLH family genes and the functional characterization of CsbHLH041 in NaCl and ABA tolerance in Arabidopsis and cucumber. BMC Plant Biol..

[96] Bai G., Yang D.H., Chao P., Yao H., Fei M., Zhang Y., Chen X., Xiao B., Li F., Wang Z.Y. (2021). Genome-wide identification and expression analysis of NtbHLH gene family in tobacco (*Nicotiana tabacum* L.) and the role of NtbHLH86 in drought adaptation. Plant Divers..

[97] Xie X.B., Li S., Zhang R.F., Zhao J., Chen Y.C., Zhao Q., Yao Y.X., You C.X., Zhang X.S., Hao Y.J. (2012). The bHLH transcription factor MdbHLH3 promotes anthocyanin accumulation and fruit colouration in response to low temperature in apples. Plant Cell Environ..

[98] Zou Q., Xu H., Yang G., Yu L., Jiang H., Mao Z., Hu J., Zhang Z., Wang N., Chen X. (2021). MdbHLH106-like transcription factor enhances apple salt tolerance by upregulating MdNHX1 expression. Plant Cell Tissue Organ Cult..

[99] Zhao Q., Fan Z., Qiu L., Che Q., Wang T., Li Y., Wang Y. (2020). MdbHLH130, an Apple bHLH Transcription Factor, Confers Water Stress Resistance by Regulating Stomatal Closure and ROS Homeostasis in Transgenic Tobacco. Front. Plant Sci..

[100] Jin C., Huang X.-S., Li K.-Q., Yin H., Li L.-T., Yao Z.-H., Zhang S.-L. (2016). Overexpression of a bHLH1 Transcription Factor of Pyrus ussuriensis Confers Enhanced Cold Tolerance and Increases Expression of Stress-Responsive Genes. Front. Plant Sci..

[101] Jiang Y., Yang B., Deyholos M.K. (2009). Functional characterization of the *Arabidopsis* bHLH92 transcription factor in abiotic stress. Mol. Genet. Genom..

[102] Babitha K.C., Ramu S.V., Pruthvi V., Mahesh P., Nataraja K.N., Udayakumar M. (2013). Co-expression of *AtbHLH17* and *AtWRKY28* confers resistance to abiotic stress in *Arabidopsis*. Transgenic Res..

[103] Yao P., Sun Z., Li C., Zhao X., Li M., Deng R., Huang Y., Zhao H., Chen H., Wu Q. (2018). Overexpression of Fagopyrum tataricum FtbHLH2 enhances tolerance to cold stress in transgenic *Arabidopsis*. Plant Physiol. Biochem..

[104] Wang R., Zhao P., Kong N., Lu R., Pei Y., Huang C., Ma H., Chen Q. (2018). Genome-Wide Identification and Characterization of the Potato bHLH Transcription Factor Family. Genes.

[105] Lin Y., Zheng H., Zhang Q., Liu C., Zhang Z. (2014). Functional profiling of EcaICE1 transcription factor gene from Eucalyptus camaldulensis involved in cold response in tobacco plants. J. Plant Biochem. Biotechnol..

[106] Zhai Y., Zhang L., Xia C., Fu S., Zhao G., Jia J., Kong X. (2016). The wheat transcription factor, TabHLH39, improves tolerance to multiple abiotic stressors in transgenic plants. Biochem. Biophys. Res. Commun..

[107] Kim J., Kim H.-Y. (2006). Functional analysis of a calcium-binding transcription factor involved in plant salt stress signaling. FEBS Lett..

[108] Li L., Gao W., Peng Q., Zhou B., Kong Q., Ying Y., Shou H. (2018). Two soybean bHLH factors regulate response to iron deficiency. J. Integr. Plant Biol..

[109] Gao Y., Jiang W., Dai Y., Xiaoyi T., Zhang C., Li H., Lu Y., Wu M., Tao X., Deng D. (2015). A maize phytochrome-interacting factor 3 improves drought and salt stress tolerance in rice. Plant Mol. Biol..

[110] Wang L., Ying Y., Narsai R., Ye L., Zheng L., Tian J., Whelan J., Shou H. (2013). Identification of OsbHLH133 as a regulator of iron distribution between roots and shoots in *Oryza sativa*. Plant Cell Environ..

[111] Zhang H., Li Y., Yao X., Liang G., Yu D. (2017). Positive Regulator of Iron Homeostasis1, OsPRI1, Facilitates Iron Homeostasis. Plant Physiol..

[112] Lei R., Li Y., Cai Y., Li C., Pu M., Lu C., Yang Y., Liang G. (2020). bHLH121 Functions as a Direct Link that Facilitates the Activation of FIT by bHLH IVc Transcription Factors for Maintaining Fe Homeostasis in *Arabidopsis*. Mol. Plant.

[113] Ahmad A., Niwa Y., Goto S., Ogawa T., Shimizu M., Suzuki A., Kobayashi K., Kobayashi H. (2015). bHLH106 Integrates Functions of Multiple Genes through Their G-Box to Confer Salt Tolerance on *Arabidopsis*. PLoS ONE.

[114] Tanabe N., Noshi M., Mori D., Nozawa K., Tamoi M., Shigeoka S. (2019). The basic helix-loop-helix transcription factor, bHLH11 functions in the iron-uptake system in *Arabidopsis thaliana*. J. Plant Res..

[115] Kiribuchi K., Jikumaru Y., Kaku H., Minami E., Hasegawa M., Kodama O., Seto H., Okada K., Nojiri H., Yamane H. (2005). Involvement of the basic helix-loop-helix tran-scription factor RERJ1 in wounding and drought stress responses in rice plants. Biosci. Biotechnol. Biochem..

[116] Chen H.C., Hsieh-Feng V., Liao P.C., Cheng W.H., Liu L.Y., Yang Y.W., Lai M.H., Chang M.C. (2017). The function of OsbHLH068 is partially redundant with its homolog, AtbHLH112, in the regulation of the salt stress response but has opposite functions to control flowering in Arabidopsis. Plant Mol. Biol..

[117] Wang F., Zhu H., Chen D., Li Z., Peng R., Yao Q. (2016). A grape bHLH transcription factor gene, VvbHLH1, increases the accumulation of flavonoids and enhances salt and drought tolerance in transgenic Arabidopsis thaliana. Plant Cell Tissue Organ Cult..

[118] Farooq M., Wahid A., Kobayashi N., Fujita D., Basra S.M.A. (2011). Plant drought stress: Effects, mechanisms and management. Agron. Sustain. Dev..

[119] Cutler S.R., Rodriguez P.L., Finkelstein R.R., Abrams S.R. (2010). Abscisic acid: Emergence of a core signaling network. Annu. Rev. Plant Biol..

[120] Nakashima K., Yamaguchi-Shinozaki K., Shinozaki K. (2014). The transcriptional regulatory network in the drought response and its crosstalk in abiotic stress responses including drought, cold, and heat. Front. Plant Sci..

[121] Takahashi F., Kuromori T., Sato H., Shinozaki K. (2018). Regulatory Gene Networks in Drought Stress Responses and Resistance in Plants. Survival Strategies in Extreme Cold and Desiccation.

[122] Yamaguchi-Shinozaki K., Shinozaki K. (1993). The plant hormone abscisic acid mediates the drought-induced expression but not the seed-specific expression of rd22, a gene responsive to dehydration stress in *Arabidopsis thaliana*. MGG Mol. Gen. Genet..

[123] Umezawa T., Okamoto M., Kushiro T., Nambara E., Oono Y., Seki M., Kobayashi M., Koshiba T., Kamiya Y., Shinozaki K. (2006). CYP707A3, a major ABA 8′-hydroxylase involved in dehydration and rehydration response in Arabidopsis thaliana. Plant J..

[124] Kim H., Seomun S., Yoon Y., Jang G. (2021). Jasmonic Acid in Plant Abiotic Stress Tolerance and Interaction with Abscisic Acid. Agronomy.

[125] Funck D., Baumgarten L., Stift M., von Wirén N., Schönemann L. (2020). Differential Contribution of P5CS Isoforms to Stress Tolerance in *Arabidopsis*. Front. Plant Sci..

[126] Verma D., Jalmi S., Bhagat P.K., Verma N., Sinha A.K. (2020). A bHLH transcription factor, MYC2, imparts salt intolerance by regulating proline biosynthesis in *Arabidopsis*. FEBS J..

[127] Pearce R.S. (2001). Plant Freezing and Damage. Ann. Bot..

[128] Stockinger E.J., Gilmour S.J., Thomashow M.F. (1997). Arabidopsis thaliana CBF1 encodes an AP2 domain-containing transcriptional activator that binds to the C-repeat/DRE, a cis-acting DNA regulatory element that stimulates transcription in response to low temperature and water deficit. Proc. Natl. Acad. Sci. USA.

[129] Gilmour S.J., Fowler S.G., Thomashow M.F. (2004). Arabidopsis transcriptional activators CBF1, CBF2, and CBF3 have matching functional activities. Plant Mol. Biol..

[130] Gilmour S.J., Zarka D.G., Stockinger E.J., Salazar M.P., Houghton J.M., Thomashow M.F. (1998). Low temperature regulation of the Arabidopsis CBF family of AP2 transcriptional activators as an early step in cold-induced COR gene expression. Plant J..

[131] Chen C.-C., Liang C.-S., Kao A.-L., Yang C.-C. (2010). HHP1, a novel signalling component in the cross-talk between the cold and osmotic signalling pathways in Arabidopsis. J. Exp. Bot..

[132] Lee H., Xiong L., Gong Z., Ishitani M., Stevenson B., Zhu J.-K. (2001). The *Arabidopsis HOS1* gene negatively regulates cold signal transduction and encodes a RING finger protein that displays cold-regulated nucleo–cytoplasmic partitioning. Genes Dev..

[133] Dong C.-H., Agarwal M., Zhang Y., Xie Q., Zhu J.-K. (2006). The negative regulator of plant cold responses, HOS1, is a RING E3 ligase that mediates the ubiquitination and degradation of ICE1. Proc. Natl. Acad. Sci. USA.

[134] Ding Y., Li H., Zhang X., Xie Q., Gong Z., Yang S. (2015). OST1 kinase modulates freezing tolerance by enhancing ICE1 stability in arabidopsis. Dev. Cell.

[135] Teige M., Scheikl E., Eulgem T., Doczi R., Ichimura K., Shinozaki K., Dangl J.L., Hirt H. (2004). The MKK2 pathway mediates cold and salt stress signaling in Arabidopsis. Mol. Cell.

[136] Li H., Ding Y., Shi Y., Zhang X., Zhang S., Gong Z., Yang S. (2017). MPK3- and MPK6-Mediated ICE1 Phosphorylation Negatively Regulates ICE1 Stability and Freezing Tolerance in Arabidopsis. Dev. Cell.

[137] Zhang Z., Li J., Li F., Liu H., Yang W., Chong K., Xu Y. (2017). OsMAPK3 Phosphorylates OsbHLH002/OsICE1 and Inhibits Its Ubiquitination to Activate OsTPP1 and Enhances Rice Chilling Tolerance. Dev. Cell.

[138] Santi S., Schmidt W. (2009). Dissecting iron deficiency-induced proton extrusion in Arabidopsis roots. New Phytol..

[139] Robinson N.J., Procter C.M., Connolly E.L., Guerinot M.L. (1999). A ferric-chelate reductase for iron uptake from soils. Nature.

[140] Eide D., Broderius M., Fett J., Guerinot M.L. (1996). A novel iron-regulated metal transporter from plants identified by functional expression in yeast. Proc. Natl. Acad. Sci. USA.

[141] Wang H.-Y., Klatte M., Jakoby M., Bäumlein H., Weisshaar B., Bauer P. (2007). Iron deficiency-mediated stress regulation of four subgroup Ib BHLH genes in Arabidopsis thaliana. Planta.

[142] Wang N., Cui Y., Liu Y., Fan H., Du J., Huang Z., Yuan Y., Wu H., Ling H.-Q. (2013). Requirement and Functional Redundancy of Ib Subgroup bHLH Proteins for Iron Deficiency Responses and Uptake in Arabidopsis thaliana. Mol. Plant.

[143] Zhang J., Liu B., Li M., Feng D., Jin H., Wang P., Liu J., Xiong F., Wang J., Wang H.-B. (2015). The bHLH Transcription Factor bHLH104 Interacts with IAA-LEUCINE RESISTANT3 and Modulates Iron Homeostasis in Arabidopsis. Plant Cell.

[144] Higuchi K., Suzuki K., Nakanishi H., Yamaguchi H., Nishizawa N.-K., Mori S. (1999). Cloning of Nicotianamine Synthase Genes, Novel Genes Involved in the Biosynthesis of Phytosiderophores. Plant Physiol..

[145] Kobayashi T., Ogo Y., Itai R.N., Nakanishi H., Takahashi M., Mori S., Nishizawa N.K. (2007). The transcription factor IDEF1 regulates the response to and tolerance of iron deficiency in plants. Proc. Natl. Acad. Sci. USA.

[146] Kobayashi T., Ogo Y., Aung M.S., Nozoye T., Itai R.N., Nakanishi H., Yamakawa T., Nishizawa N.K. (2010). The spatial expression and regulation of transcription factors IDEF1 and IDEF2. Ann. Bot..

[147] Lindermayr C., Durner J. (2009). S-Nitrosylation in plants: Pattern and function. J. Proteom..

[148] Barberon M., Zelazny E., Robert S., Conéjéro G., Curie C., Friml J., Vert G. (2011). Monoubiquitin-dependent endocytosis of the Iron-Regulated Transporter 1 (IRT1) transporter controls iron uptake in plants. Proc. Natl. Acad. Sci. USA.

[149] Shin L.J., Lo J.C., Chen G.H., Callis J., Fu H., Yeh K.C. (2013). IRT1 degradation factor1, a ring E3 Ubiquitin ligase, regulates the degradation of iron-regulated transporter1 in Arabidopsis. Plant Cell.

[150] Matthiadis A., Long T.A. (2016). Further insight into BRUTUS domain composition and functionality. Plant Signal. Behav..

[151] Qian Y.C., Zhang T.Y., Yu Y., Gou L.P., Yang J.T., Xu J., Pi E.X. (2021). Regulatory Mechanisms of bHLH Transcription Factors in Plant Adaptive Responses to Various Abiotic Stresses. Front. Plant Sci..

